# Riboflavin did not provide anti-inflammatory or antioxidant effects in an experimental model of sepsis

**DOI:** 10.1590/1414-431X2022e12107

**Published:** 2022-05-27

**Authors:** A.H.F. Vale, D.C. Nascimento, A.R. Pineros, R.G. Ferreira, J.D. Santos, D.C. Aragon, F.Q. Cunha, F.S. Ramalho, J.C. Alves-Filho, A.P.C.P. Carlotti

**Affiliations:** 1 Departamento de Puericultura e Pediatria, Faculdade de Medicina de Ribeirão Preto, Universidade de São Paulo, Ribeirão Preto, SP, Brasil; 2 Departamento de Farmacologia, Faculdade de Medicina de Ribeirão Preto, Universidade de São Paulo, Ribeirão Preto, SP, Brasil; 3 Department of Pediatrics, Indiana University School of Medicine, Indianapolis, IN, USA; 4 Departamento de Patologia, Faculdade de Medicina de Ribeirão Preto, Universidade de São Paulo, Ribeirão Preto, SP, Brasil

**Keywords:** Sepsis, Riboflavin, Inflammatory response, Oxidative stress, Organ dysfunction

## Abstract

We aimed to evaluate whether the administration of riboflavin to septic animals reduces inflammation, oxidative stress, organ dysfunction, and mortality. C57BL/6 mice, 6-8 weeks old, were allocated to the study group (polymicrobial sepsis induced by cecal ligation and puncture (CLP) + antibiotic + *iv* riboflavin), control (CLP + antibiotic + *iv* saline), or naïve (non-operated controls). Serum concentrations of alanine aminotransferase (ALT), creatine kinase-MB (CK-MB), urea, and creatinine, and markers of inflammation [interleukin (IL)-6, tumor necrosis factor (TNF)-α, keratinocyte-derived chemokine (KC), and macrophage inflammatory protein (MIP)-2)], and oxidative stress (malondialdehyde (MDA) were measured 12 h after the experiment. Animal survival rates were calculated after 7 days. Means between groups were compared using linear regression models adjusted under the Bayesian approach. No significant difference was observed between control and study groups in serum concentrations of IL-6 (95% credible interval) (-0.35 to 0.44), TNF-α (-15.7 to 99.1), KC (-0.13 to 0.05), MIP-2 (-0.84 to 0.06), MDA (-1.25 to 2.53), or ALT (-6.6 to 11.5). Serum concentrations of CK-MB (-145.1 to -30.1), urea (-114.7 to -15.1), and creatinine (-1.14 to -0.01) were higher in the study group. Survival was similar in both groups (P=0.8). Therefore, the use of riboflavin in mice undergoing sepsis induced by CLP did not reduce inflammation, oxidative stress, organ dysfunction, or mortality compared with placebo.

## Introduction

According to the Third International Consensus Definitions for Sepsis and Septic Shock (Sepsis-3), sepsis is defined as life-threatening organ dysfunction caused by a dysregulated host response to infection (1). It is a global health issue, with increasing incidence, high mortality rates, and substantial treatment costs in developed and developing countries (2-4). Currently, there is no specific therapy for sepsis.

Pre-clinical animal studies are important for the development of new treatments for sepsis. However, their applicability may be limited because of the limitations of the models and heterogeneity of human disease (5). The cecal ligation and puncture (CLP) is the most frequently used model because it can partially reproduce the progression of the pathophysiological phenomena observed in humans. It induces sepsis by promoting leakage of microbial flora into the peritoneum, which results in peritonitis, followed by polymicrobial translocation into the bloodstream, activation of the inflammatory response, and organ dysfunction (6-8).

The pathogenesis of sepsis-induced organ dysfunction is not completely understood (9). Immune response alterations, including cytokine storm, excessive release of nitric oxide, and oxidative stress, may cause tissue damage and organ dysfunction. In addition, mitochondrial injury and altered cellular bioenergetics may also be involved (10).

Riboflavin (vitamin B-2) is a water-soluble vitamin, essential for nutrient metabolism and normal cell function. It acts as a precursor of flavin mononucleotide (FMN) and flavin adenine dinucleotide (FAD), which are crucial for mitochondrial energy production mediated by the electron transport chain (11). Riboflavin also plays a key role in the conversion of oxidized glutathione to its reduced form, which has antioxidant properties, especially against lipid peroxidation. In addition, riboflavin can affect the activity of antioxidant enzymes, such as glutathione peroxidase, superoxide dismutase, and catalase. Experimental and human studies have demonstrated that riboflavin can attenuate reperfusion oxidative injury, probably through its activity as a free radical scavenger (12,13). In experimental models of sepsis induced by lipopolysaccharide (LPS) injection, riboflavin reduced serum levels of inflammatory cytokines and nitric oxide (14-16). High doses of riboflavin (100 to 400 mg/d) have been used in adults and pediatric patients with migraine and mitochondrial diseases, with no reports of serious toxicity (17,18).

As riboflavin is a low-cost, safe, and well-tolerated vitamin with anti-inflammatory and antioxidant effects, the aim of this study was to evaluate whether the administration of riboflavin to an experimental model of polymicrobial sepsis induced by CLP reduces inflammation, oxidative stress, organ dysfunction, and mortality compared with placebo.

## Material and Methods

### Mice

Male C57BL/6 mice (wild-type, WT) aged 6 to 8 weeks and weighing 18 to 25 g were obtained from the animal facility of Ribeirão Preto Medical School, University of São Paulo. The animals were housed in barrier cages under controlled environmental conditions (12 h light/dark cycles, 55±5% humidity, 23-25°C) and received water and food *ad libitum*. The study was approved by the Animal Ethics Committee of Ribeirão Preto Medical School, University of São Paulo (Protocol number 009/2016).

### Drugs

The following drugs were used: ertapenem sodium (Merck Research Laboratory, USA), riboflavin (riboflavin 5'-sodium monophosphate; Sigma-Aldrich Laboratories, USA), xylazine, and ketamine (União Química, Brazil). Ertapenem sodium and riboflavin were diluted in 0.9% saline. Riboflavin was handled with light protection.

### Animal model of sepsis induced by CLP

Sepsis was induced by CLP, as described in detail elsewhere (7). Briefly, mice were anesthetized intraperitoneally with ketamine (100 mg/kg) and xylazine (10 mg/kg), and a midline incision was performed in the abdomen. The cecum was exposed and ligated below the ileo-cecal valve and was perforated twice with a sterile 18-gauge needle. The cecum was then gently squeezed to extrude a small amount of feces from the perforations and subsequently returned to the peritoneal cavity (mid-grade sepsis). All animals received 1 mL of saline subcutaneously immediately after surgery.

### Experimental protocol

**Figure 1:**
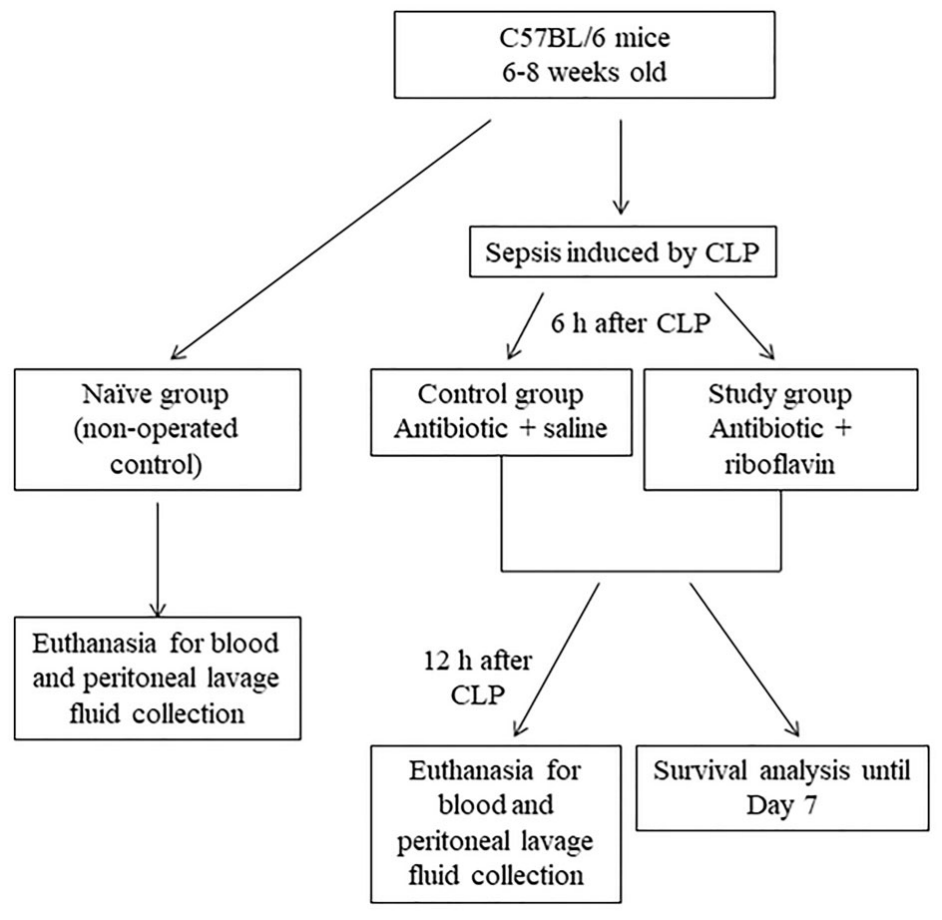
Flow diagram of the study. CLP: cecal ligation and puncture.

C57BL/6 mice were randomly allocated to three groups: study group, control group, or naïve group. Mice in the study and control groups underwent CLP and received ertapenem sodium (30 mg/kg diluted in 200 µL of 0.9% saline) as a single dose administered intraperitoneally, 6 h after surgery. In addition to antibiotic therapy, mice in the study group also received riboflavin (20 mg/kg diluted in 200 µL of 0.9% saline), while animals in the control group received placebo (200 µL of 0.9% saline), both administered as a single dose via retroocular venous plexus, 6 h after surgery, under inhalation anesthesia with 2% isoflurane. Mice in the naïve group (non-operated control) did not receive antibiotics, riboflavin, or placebo. This group was used as a control of the surgical procedure, because it has been shown that in protocols involving CLP-induced sepsis, there is no difference in the inflammatory and immune responses between naïve and sham-operated mice (19). There were 5 to 7 animals in each group and all experiments were performed in duplicate. Figure 1 shows the flow diagram of the study.

Twelve hours after the beginning of the experiments, mice were euthanized with a lethal dose of anesthetics. Peritoneal lavage fluid and blood samples were collected for quantification of bacteria, assessment of neutrophil migration to the peritoneal cavity, and measurement of markers of organ dysfunction, inflammation, and oxidative stress (Supplementary Figure S1).

#### Quantification of bacteria

The quantification of bacteria in blood and peritoneal lavage fluid was determined as previously described (20). In brief, peritoneal lavage fluid and blood samples were plated on Mueller-Hinton agar dishes (Difco Laboratories, USA) and incubated for 18 h at 37°C. The number of colony-forming units (CFU) was quantified, and the results are reported as log CFU/mL.

#### Neutrophil migration to the peritoneal cavity

Cells from the peritoneal cavity were collected by injecting 2 mL of phosphate buffered saline (PBS) containing EDTA (1 nM), and the total number of cells was counted using a Neubauer chamber. Differential cell counts were performed in peritoneal lavage aliquots, which were centrifuged at 130 *g* at room temperature for 7 min (Cytospin 3, Shandon Southern Products, UK). Cells were subsequently fixed on glass slides and then stained with Rapid Panotic dye (Laborclin, Brazil). The results are reported as the number of neutrophils in peritoneal cavity.

#### Characterization of organ dysfunction

Sepsis-induced organ dysfunction was assessed by serum concentrations of aspartate aminotransferase (AST), alanine aminotransferase (ALT), creatine kinase-MB isoenzyme (CK-MB), urea, and creatinine. Concentrations of AST, ALT, CK-MB, and urea were determined using an analytical kit (Labtest, Brazil), and creatinine concentration was measured by a colorimetric method (Bioclin, Brazil).

#### Biomarkers of inflammation

Enzyme-linked immunosorbent assay (ELISA) was used to measure serum and peritoneal lavage fluid concentrations of interleukin (IL)-6, tumor necrosis factor (TNF)-α, keratinocyte-derived chemokine (KC) (eBioscience Inc., USA), and macrophage inflammatory protein (MIP)-2 (R&D Systems, Inc., USA) according to manufacturers’ instructions.

#### Assessment of oxidative stress

Oxidative stress occurs when there is increased production of reactive oxygen species or impairment in the antioxidant systems. However, direct measurement of reactive oxygen species in biological samples is difficult due to their short half-life and rapid reactivity. Hence, oxidative stress is usually assessed by the measurement of products of oxidation of cell components, such as lipids. Malondialdehyde (MDA) is formed during peroxidation of polyunsaturated fatty acids, as a product of free radical generation, and is a marker of oxidative damage to cell membranes. Thus, MDA was used to estimate oxidative stress. Serum concentrations of MDA were measured using the thiobarbituric acid reactive substances (TBARS) method (21,22).

#### Survival analysis

The animals were monitored twice daily and the survival rate was assessed for 7 days. At the end of this period, mice that survived were euthanized with an anesthetic overdose.

### Statistical analysis

Analysis was carried out using OpenBUGS software (Free Software Foundation, Inc., USA) and the JAGS package of the R 3.5.1 software (R Foundation for Statistical Computing, Austria). Group means were compared using linear regression models, which were adjusted under the Bayesian approach, obtaining estimates of differences between the means and 95% credible intervals (95%CrI). When all observations in a group were zero, it was not possible to perform data analysis. Survival analysis was performed using the Kaplan-Meier method, and statistical differences between curves were assessed using the log-rank test.

## Results

**Figure 2:**
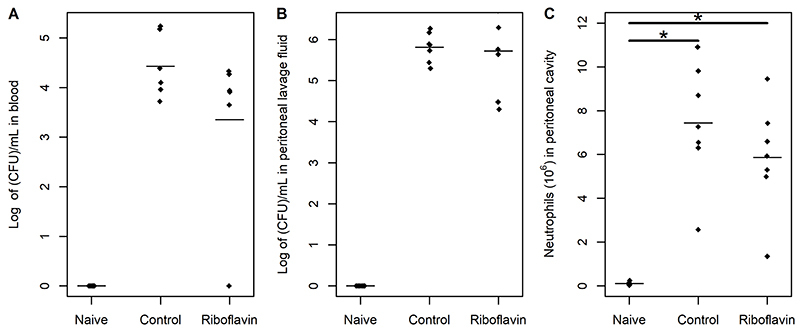
Quantification of bacteria reported as log of colony-forming units (CFU)/mL in blood (**A**) and peritoneal lavage fluid (**B**) of naïve, control, and study groups (n=5 to 7 animals per group). **C**, Quantification of neutrophils reported as number of neutrophils ×10^6^ in peritoneal cavity of naïve, control, and study groups (n=7 animals per group). The horizontal bars represent means. *Significant difference between groups (linear regression models adjusted under the Bayesian approach).

There was no significant difference between control and study groups in bacterial counts in blood (95%CrI: -0.34 to 2.87) or peritoneal lavage fluid (95%CrI: -0.9 to 0.85) (Figure 2A and B). Also, there was no significant difference between control and study groups in neutrophil migration to the peritoneal cavity (96% CrI -0.74 to 4.26). However, the number of neutrophils in the peritoneal cavity was greater in controls (95%CrI: -0.97 to -5.04) and the study group (95%CrI: -8.2 to -3.4) compared to the naïve group (Figure 2C).

**Figure 3:**
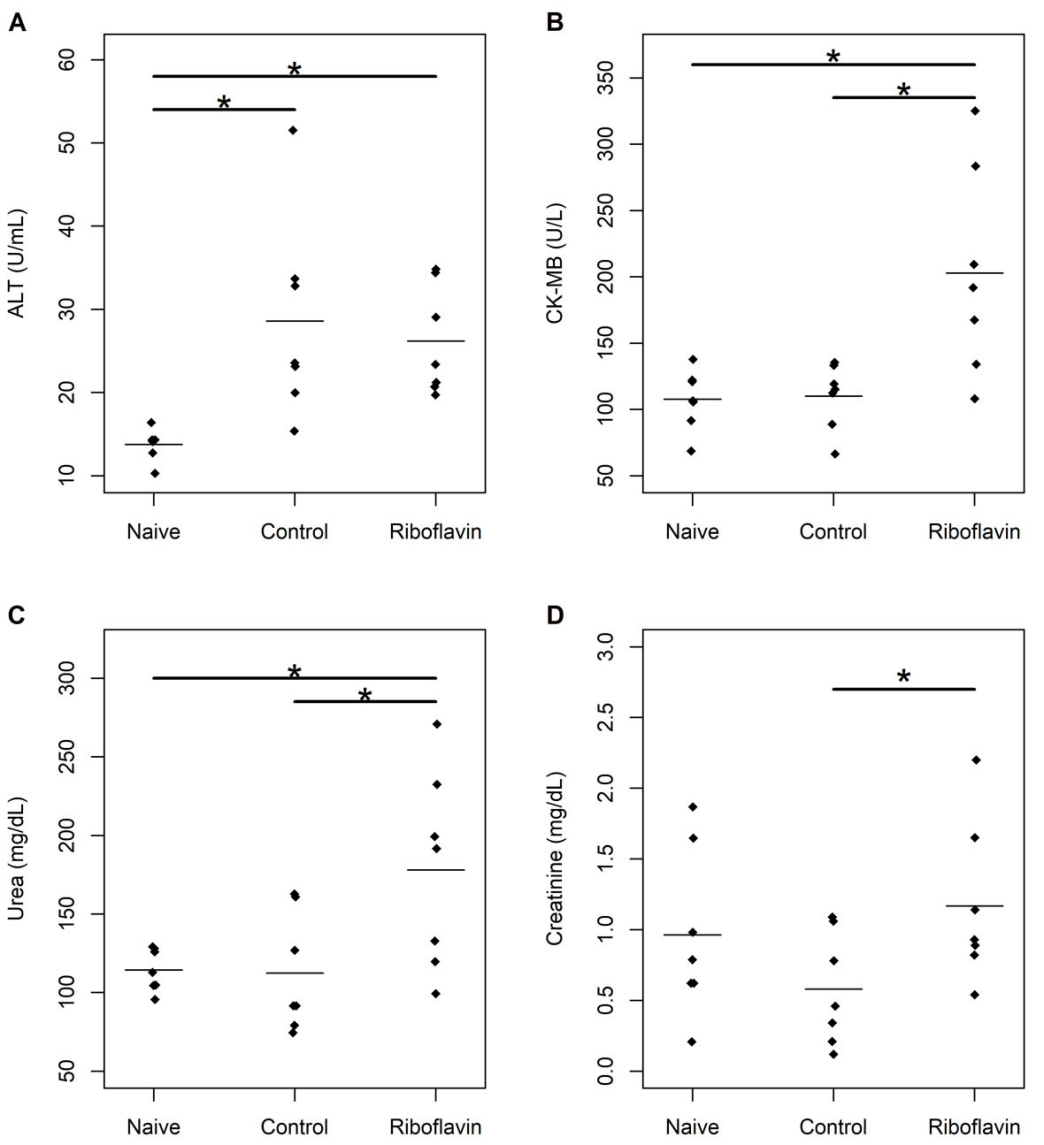
Serum concentrations of biomarkers of organ dysfunction in naïve, control, and study groups (n=7 animals per group). **A**, Alanine aminotransferase (ALT); **B**, creatine kinase-MB isoenzyme (CK-MB); **C**, urea; **D**, creatinine. The horizontal bars represent means. *Significant difference between groups (linear regression models adjusted under the Bayesian approach).

No significant difference was observed between control and study groups in serum concentrations of ALT (95%CrI: -6.6 to 11.5); nevertheless, they were significantly higher in the control (95%CrI -23.4 to -5.8) and study groups (95%CrI: -20.9 to -3.6) compared to the naïve group. Serum concentrations of CK-MB were significantly higher in the study group compared to naïve (95%CrI: -144.4 to -37.5) and control groups (95%CrI: -145.1 to -30.1). Moreover, serum concentrations of urea were significantly higher in the study group compared to naïve (95%CrI: -112.8 to -12.9) and control groups (95%CrI: -114.7 to -15.1). The study group also had higher serum concentrations of creatinine compared to the control group (95%CrI: -1.14 to -0.01) (Figure 3).

**Figure 4:**
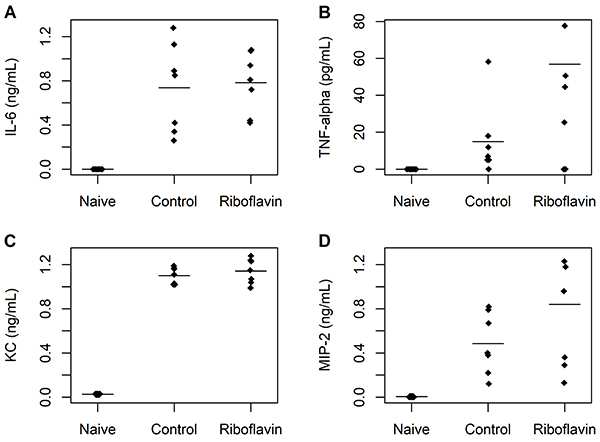
Serum concentrations of biomarkers of inflammation in naïve, control, and study groups (n=7 animals per group). **A**, Interleukin (IL)-6; **B**, tumor necrosis factor (TNF)-alpha; **C**, keratinocyte-derived chemokine (KC); **D**, macrophage inflammatory protein (MIP)-2. The horizontal bars represent means. There were no significant differences between control and study groups.

**Figure 5:**
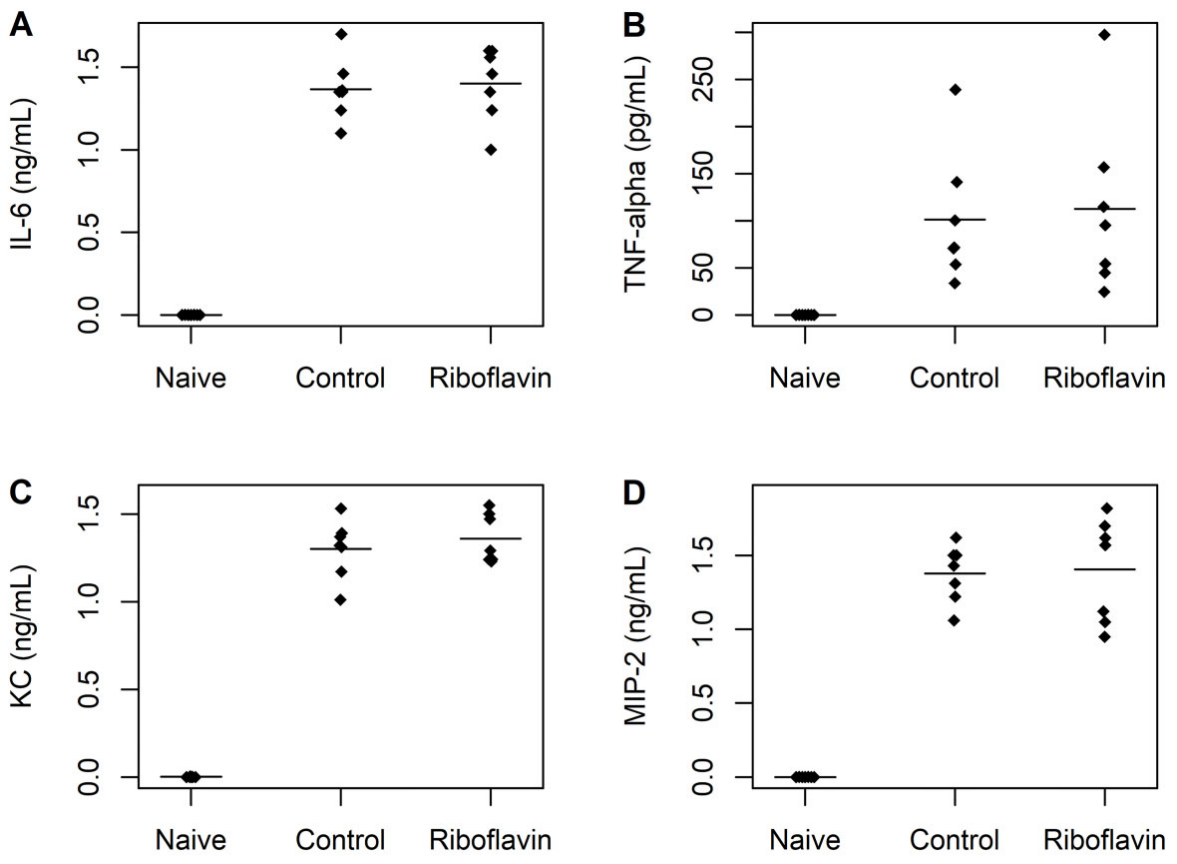
Concentrations of biomarkers of inflammation in peritoneal lavage fluid in naïve, control, and study groups (n=7 animals per group). **A**, Interleukin (IL)-6; **B**, tumor necrosis factor (TNF)-alpha; **C**, keratinocyte-derived chemokine (KC); **D**, macrophage inflammatory protein (MIP)-2. The horizontal bars represent means. There were no significant differences between control and study groups.

There were no significant differences between control and study groups in serum concentrations of IL-6 (95%CrI: -0.35 to 0.44), TNF-α (95%CrI: -15.7 to 99.1), KC (95%CrI: -0.13 to 0.05), or MIP-2 (95%CrI: -0.84 to 0.06) (Figure 4). In addition, no significant differences between control and study groups were observed in peritoneal lavage fluid concentrations of IL-6 (95%CrI: -0.19 to 0.27), TNF-α (95%CrI: -79.8 to 104.9), KC (95%CrI: -0.2 to 0.08), or MIP-2 (95%CrI: -0.31 to 0.37) (Figure 5).

**Figure 6:**
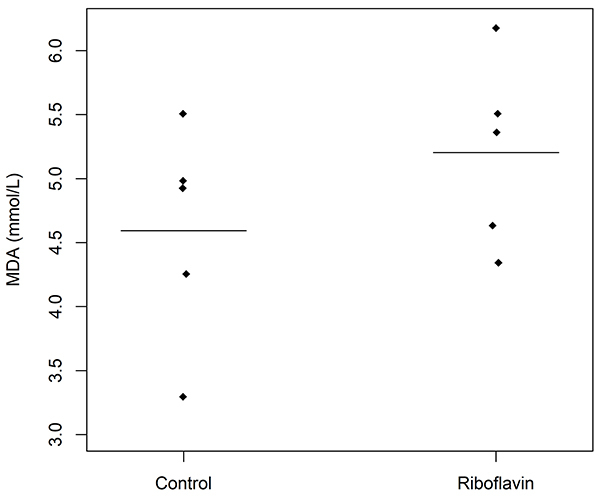
Serum concentrations of malondialdehyde (MDA) (mM) in control and study groups (n=5 animals per group). The horizontal bars represent means. There were no significant differences between control and study groups.

**Figure 7:**
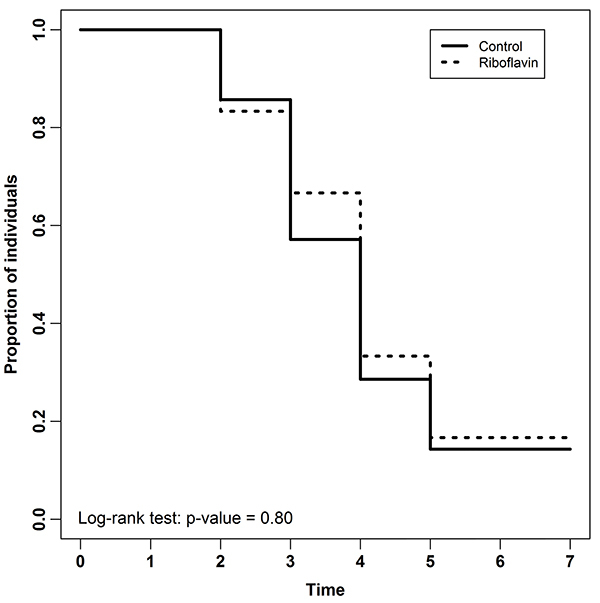
Kaplan-Meier curves for survival analysis of C57BL/6 mice submitted to sepsis induced by cecal ligation and puncture (n= 7 animals per group).

No significant difference was observed between control and study groups in serum concentrations of MDA (95%CrI: -1.25 to 2.53) (Figure 6). Survival was also similar in both groups (P=0.8) (Figure 7).

## Discussion

In the present study, an experimental model of CLP was used to assess whether the administration of riboflavin to septic mice resulted in reduction of inflammation, oxidative stress, organ dysfunction, and mortality compared with placebo. Our data showed that 12 h after the surgical procedure the animals had sepsis, since we observed bacterial growth in blood and increased serum concentrations of the inflammatory markers IL-6, TNF-α, KC, and MIP-2 in mice submitted to CLP compared to naïve. However, there was no reduction in bacterial counts in blood or peritoneal lavage fluid in mice treated with riboflavin 6 h after the procedure compared to septic animals in the control group. Also, treatment with riboflavin did not affect neutrophil migration to the peritoneal cavity. In contrast, a previous publication showed a significant reduction in bacterial counts in blood of mice that received a bolus dose of riboflavin (20 mg/kg) 24 h before the intravenous inoculation of *Escherichia coli* (15). However, the therapeutic intervention was administered before the septic insult, which precludes clinical applicability of these findings (23). Furthermore, studies based on intraperitoneal or intravenous bacterial injection do not mimic human sepsis, which usually has a continuous reservoir for bacteria release (8,24). In this context, the CLP model provides a better representation of human sepsis compared with bacterial injection (6).

To assess whether treatment with riboflavin promoted an anti-inflammatory action, with a consequent reduction in the damage generated by the dysregulated response of the immune system during sepsis, we performed the quantification of the pro-inflammatory cytokines IL-6 and TNF-α and the chemokines KC and MIP-2 in serum and peritoneal lavage fluid of septic mice. We found no significant differences between study and control groups in serum and peritoneal lavage fluid concentrations of inflammatory mediators. Therefore, riboflavin did not have a local or systemic anti-inflammatory effect in our study. This finding is in accordance with the neutrophil migration pattern observed in the present study, since the chemokines KC and MIP-2 play an important role in the recruitment of neutrophils to the infection site, being considered functional homologs of IL-8 in mice (25). Our data disagree with those of previous studies of LPS-induced sepsis that found decreased plasma concentrations of IL-6, TNF-α, and MIP-2 in mice treated with riboflavin 6 h after LPS injection (14-16). This discrepancy may be due to the important differences inherent to the experimental models (8). Previous studies comparing models of sepsis induced by LPS injection with CLP-induced sepsis demonstrate that the inflammatory mediators such as IL-6, TNF-α, KC, and MIP-2 show different time profiles. Their peak concentrations are greater, they have a shorter duration and occur earlier in the LPS model in contrast to the lower and more prolonged release of cytokines in the CLP model, which is similar to what occurs in human sepsis (8,26,27).

In the present study, markers of organ dysfunction were assessed 12 h after CLP because organ dysfunction usually occurs 6 to 12 h after sepsis initiation (28,29). Previous studies showed increased concentrations of AST, ALT, CK-MB, urea, and creatinine in CLP-induced sepsis 8 to 12 h after the septic insult (29,30). In our study, riboflavin did not reduce the concentrations of markers of organ dysfunction. In fact, CK-MB, urea, and creatinine were even higher in mice treated with riboflavin. Thus, considering that inflammatory response is one of the mechanisms that contribute to organic injury (31), the fact that riboflavin treatment did not result in a reduction in the concentrations of pro-inflammatory mediators in septic mice could justify, in part, the lack of improvement in most markers of organ dysfunction.

It has been consistently demonstrated that, during sepsis, cellular energy failure related to mitochondrial dysfunction, resulting from oxidative stress, is associated with a worse outcome in critically ill patients (32-34). However, we did not observe a reduction in MDA levels in septic animals treated with riboflavin. The lack of an antioxidant effect in our study could be due to insufficient amounts of riboflavin reaching the mitochondria, since the desired effect of a medicine targeting the mitochondria can be achieved only if the bioactive molecule is absorbed by the organ and/or target cell and accumulated in the subcellular site (35). There is evidence in humans that oxidative stress along with inflammation is involved in the pathways that are activated during sepsis leading to organ dysfunction and death. Indeed, in septic neonates, there is a significant increase in circulating concentrations of TNF-α and MDA, compared to healthy controls (36).

In our study, riboflavin had no benefit in survival. Our results diverged from previous studies, which demonstrated increased survival in mice treated with riboflavin after LPS injection or as a pre-treatment before the injection of *Escherichia coli* (14,15). However, we must be cautious when comparing research carried out using very different methodologies. The Minimum Quality Threshold in Pre-Clinical Sepsis Studies (MQTiPSS) guidelines recommend that the therapeutic interventions should be started after the septic insult, replicating clinical care, and that the LPS model should not be used for reproducing human sepsis (23). Thus, the contrast between our results and the positive results of studies that used riboflavin (14-16) probably occurred due to the fact that the endotoxic shock induced by LPS and injection of bacteria are models that do not reproduce the complexity of human sepsis (29,37,38). These conflicting results emphasize the importance of testing treatments for sepsis in different animal models prior to proceeding to clinical trials (8). Although the CLP model is considered the gold standard for sepsis studies because it mimics the clinical course of intra-abdominal sepsis (8,29), it has several limitations, such as variation in the experimental procedure, including the length of ligated cecum, the size and number of punctures performed, the amount of stool that leaks into the peritoneal cavity, and the age, sex, and strain of the animals (24,27,29).

### Limitations of the study

The animals were randomly allocated into groups, but the study was not blinded. Moreover, there could have been variations in the CLP experimental procedure, although it was performed by highly-skilled researchers (DCN and RGF). In addition, we used fixed time intervals between the septic insult and the administration of the antibiotic and riboflavin, and we collected blood and peritoneal lavage fluid samples only 12 h after the surgical procedure. However, in humans, the magnitude or timing of immune response to the septic insult may be different, even if the intensity of the insult is kept constant (8,38,39). The strength of this study is that we showed that the use of riboflavin in an experimental model of CLP-induced sepsis did not provide the benefits reported in other studies based on the LPS model or bacteria injection. Similarly, contrasting results have also been reported in studies on TNF-α inhibitors, which initially showed impressive protection when tested in murine models of LPS-induced sepsis, but subsequent human studies have failed to show any benefit in mortality. This could have been predicted from studies using murine models of CLP-induced sepsis, which also demonstrated reduced survival with the use of TNF-α inhibitors (8,40).

In conclusion, the use of riboflavin associated with antimicrobial treatment in an experimental model of CLP-induced sepsis did not provide anti-inflammatory or antioxidant effects, did not reduce or even increased the concentrations of some organ dysfunction markers, such as CK-MB, urea, and creatinine, and did not reduce mortality.

## Supplementary Material

Click here to view [pdf].

## References

[1] Singer M, Deutschman CS, Seymour CW, Shankar-Hari M, Annane D, Bauer M (2016). The Third International Consensus Definitions for Sepsis and Septic Shock (Sepsis-3). JAMA.

[2] Gaieski DF, Edwards JM, Kallan MJ, Carr BG (2013). Benchmarking the incidence and mortality of severe sepsis in the United States. Crit Care Med.

[B03] Stoller J, Halpin L, Weis M, Aplin B, Qu W, Georgescu C (2016). Epidemiology of severe sepsis: 2008-2012. J Crit Care.

[4] Neira RAQ, Hamacher S, Japiassu AM (2018). Epidemiology of sepsis in Brazil: Incidence, lethality, costs, and other indicators for Brazilian Unified Health System hospitalizations from 2006 to 2015. PLoS One.

[5] Zingarelli B, Coopersmith CM, Drechsler S, Efron P, Marshall JC, Moldawer L (2019). Part I: minimum quality threshold in preclinical sepsis studies (MQTiPSS) for study design and humane modeling endpoints. Shock.

[6] Dejager L, Pinheiro I, Dejonckheere E, Libert C (2011). Cecal ligation and puncture: The gold standard model for polymicrobial sepsis?. Trends Microbiol.

[B07] Toscano MG, Ganea D, Gamero AM (2011). Cecal ligation puncture procedure. J Vis Exp.

[8] Lewis AJ, Seymour CW, Rosengart MR (2016). Current murine models of sepsis. Surg Infect (Larchmt).

[9] Cecconi M, Evans L, Levy M, Rhodes A (2018). Sepsis and septic shock. Lancet.

[10] Ghnewa YG, Fish M, Jennings A, Carter MJ, Shankar-Hari M (2020). Goodbye SIRS? Innate, trained and adaptive immunity and pathogenesis of organ dysfunction. Med Klin Intensivmed Notfmed.

[11] Udhayabanu T, Manole A, Rajeshwari M, Varalakshmi P, Houlden H, Ashokkumar B (2017). Riboflavin responsive mitochondrial dysfunction in neurodegenerative diseases. J Clin Med.

[12] Ashoori M, Saedisomeolia A (2014). Riboflavin (vitamin B2) and oxidative stress: a review. Br J Nutr.

[13] Sanches SC, Ramalho LN, Mendes-Braz M, Terra VA, Cecchini R, Augusto MJ (2014). Riboflavin (vitamin B-2) reduces hepatocellular injury following liver ischaemia and reperfusion in mice. Food Chem Toxicol.

[14] Toyosawa T, Suzuki M, Kodama K, Araki S (2004). Effects of intravenous infusion of highly purified vitamin B2 on lipopolysaccharide-induced shock and bacterial infection in mice. Eur J Pharmacol.

[B15] Toyosawa T, Suzuki M, Kodama K, Araki S (2004). Highly purified vitamin B2 presents a promising therapeutic strategy for sepsis and septic shock. Infect Immun.

[16] Kodama K, Suzuki M, Toyosawa T, Araki S (2005). Inhibitory mechanisms of highly purified vitamin B2 on the productions of proinflammatory cytokine and NO in endotoxin-induced shock in mice. Life Sci.

[17] Condò M, Posar A, Arbizzani A, Parmeggiani A (2009). Riboflavin prophylaxis in pediatric and adolescent migraine. J Headache Pain.

[18] Buehler BA (2011). Vitamin B2: Riboflavin. J Evid-Based Complem Altern Med.

[19] Nascimento DC, Alves-Filho JC, Sônego F, Fukada SY, Pereira MS, Benjamim C (2010). Role of regulatory T cells in long-term immune dysfunction associated with severe sepsis. Crit Care Med.

[20] Godshall CJ, Scott MJ, Peyton JC, Gardner SA, Cheadle WG (2002). Genetic background determines susceptibility during murine septic peritonitis. J Surg Res.

[21] Katerji M, Filippova M, Duerksen-Hughes P (2019). Approaches and methods to measure oxidative stress in clinical samples: research applications in the cancer field. Oxid Med Cell Longev.

[22] Manso PH, Carmona F, Dal-Pizzol F, Petronilho F, Cardoso F, Castro M (2013). Oxidative stress markers are not associated with outcomes after pediatric heart surgery. Paediatr Anaesth.

[23] Osuchowski MF, Ayala A, Bahrami S, Bauer M, Boros M, Cavaillon JM (2018). Minimum quality threshold in pre-clinical sepsis studies (MQTiPSS): an international expert consensus initiative for improvement of animal modeling in sepsis. Intensive Care Med Exp.

[24] Poli-de-Figueiredo LF, Garrido AG, Nakagawa N, Sannomiya P (2008). Experimental models of sepsis and their clinical relevance. Shock.

[25] Sônego F, Castanheira FVS, Ferreira RG, Kanashiro A, Leite CA, Nascimento DC (2016). Paradoxical roles of the neutrophil in sepsis: protective and deleterious. Front Immunol.

[26] Mera S, Tatulescu D, Cismaru C, Bondor C, Slavcovici A, Zanc V (2011). Multiplex cytokine profiling in patients with sepsis. APMIS.

[27] Libert C, Ayala A, Bauer M, Cavaillon JM, Deutschman C, Frostell C (2019). Part II: minimum quality threshold in preclinical sepsis studies (MQTiPSS) for types of infections and organ dysfunction endpoints. Shock.

[28] Angus DC, Van Der Poll T (2013). Severe sepsis and septic shock. N Engl J Med.

[29] Li JL, Li G, Jing XZ, Li YF, Ye QY, Jia HH (2018). Assessment of clinical sepsis-associated biomarkers in a septic mouse model. J Int Med Res.

[30] Liu J, Li J, Tian P, Guli B, Weng G, Li L (2019). H 2 S attenuates sepsis-induced cardiac dysfunction via a PI3K/Akt-dependent mechanism. Exp Ther Med.

[31] Sterling SA, Puskarich MA, Shapiro NI, Trzeciak S, Kline JA, Summers RL (2013). Characteristics and outcomes of patients with vasoplegic versus tissue dysoxic septic shock. Shock.

[32] Brealey D, Brand M, Hargreaves I, Heales S, Land J, Smolenski R (2002). Association between mitochondrial dysfunction and severity and outcome of septic shock. Lancet.

[33] Carré JE, Orban JC, Re L, Felsmann K, Iffert W, Bauer M (2010). Survival in critical illness is associated with early activation of mitochondrial biogenesis. Am J Respir Crit Care Med.

[34] Lorente L, Martín MM, Abreu-González P, Domínguez-Rodriguez A, Labarta L, Díaz C (2013). Sustained high serum malondialdehyde levels are associated with severity and mortality in septic patients. Crit Care.

[35] Galley HF (2010). Bench-to-bedside review: Targeting antioxidants to mitochondria in sepsis. Crit Care.

[36] Poggi C, Dani C (2018). Sepsis and oxidative stress in the newborn: From pathogenesis to novel therapeutic targets. Oxid Med Cell Longev.

[37] Song L, Zou Y, Cao Z (2018). Comparison of two different models of sepsis induced by cecal ligation and puncture in rats. J Surg Res.

[38] Rittirsch D, Hoesel LM, Ward PA (2007). The disconnect between animal models of sepsis and human sepsis. J Leukoc Biol.

[39] Lewis AJ, Yuan D, Zhang X, Angus DC, Rosengart MR, Seymour CW (2016). Use of biotelemetry to define physiology-based deterioration thresholds in a murine cecal ligation and puncture model of sepsis. Crit Care Med.

[40] Remick D, Manohar P, Bolgos G, Rodriguez J, Moldawer L, Wollenberg G (1995). Blockade of tumor necrosis factor reduces lipopolysaccharide lethality, but not the lethality of cecal ligation and puncture. Shock.

